# Influence of Visual and Vestibular Hypersensitivity on Derealization and Depersonalization in Chronic Dizziness

**DOI:** 10.3389/fneur.2019.00069

**Published:** 2019-02-13

**Authors:** Michel Toupet, Christian Van Nechel, Charlotte Hautefort, Sylvie Heuschen, Ulla Duquesne, Anne Cassoulet, Alexis Bozorg Grayeli

**Affiliations:** ^1^Otolaryngology Department, Dijon University Hospital, Université Bourgogne-Franche Comté, Dijon, France; ^2^Centre d'Explorations Fonctionnelles Otoneurologiques, Paris, France; ^3^Institut de Recherche Oto-Neurologique (IRON), Paris, France; ^4^Clinique des Vertiges, Brussels, Belgium; ^5^Otolaryngology Department, Hôpital Lariboisière, APHP, Paris, France; ^6^Le2i, Electronic, Image and Computer Research Laboratory, Dijon, France

**Keywords:** chronic vertigo, persistent postural-perceptual dizziness, migraine, optic flow vertigo, motion sickness, anxiety, depression, depersonalization/derealization disorder

## Abstract

**Objective:** The aim of this study was to investigate the relation between visual and vestibular hypersensitivity, and Depersonalization/Derealization symptoms in patients with chronic dizziness.

**Materials and Methods:** 319 adult patients with chronic dizziness for more than 3 months (214 females and 105 males, mean age: 58 years, range: 13–90) were included in this prospective cross-sectional study. Patients underwent a complete audio-vestibular workup and 3 auto questionnaires: Hospital Anxiety and Depression (HAD), Depersonalization/Derealization Inventory (DDI), and an in-house questionnaire (Dizziness in Daily Activity, DDA) assessing 9 activities with a score ranging from 0 (no difficulty) to 10 (maximal discomfort) and 11 (avoidance) to detect patients with visual and vestibular hypersensitivity (VVH, a score > 41 corresponding to mean + 1 standard deviation).

**Results:** DDI scores were higher in case of VVH (6.9 ± 6.79, *n* = 55 vs. 4.2 ± 4.81, *n* = 256 without VVH, *p* < 0.001, unpaired *t-*test), migraine (6.1 ± 6.40, *n* = 110 vs. 4.0 ± 4.42, *n* = 208no migraine, *p* < 0.001, unpaired *t-*test), and motion sickness (6.8 ± 5.93, *n* = 41 vs. 4.4 ± 5.11, *n* = 277 no motion sickness, *p* < 0.01, unpaired *t-*test). Women scored DDI higher than men (5.1 ± 5.42, *n* = 213 vs. 3.9 ± 4.91, *n* = 105, respectively, *p* < 0.05, unpaired *t-*test). DDI scores were also related to depression and anxiety. DDI score was also higher during spells than during the basal state.

**Conclusion:** During chronic dizziness, Depersonalization/Derealization symptoms seem to be related to anxiety and depression. Moreover, they were prominent in women, in those with visual and vestibular hypersensitivity, migraine, and motion sickness.

## Introduction

Chronic dizziness in patients with a unilateral stable vestibular weakness or even normal inner ear function and no neurological abnormality is a diagnostic and therapeutic challenge. In an attempt to define at least a part of this population, a syndrome designated as phobic postural vertigo was proposed to define those with a hypersensitivity to visual stimuli and movements (1). Other medical terms such as optic flow vertigo (2), chronic subjective dizziness (3), and more recently persistent postural-perceptual dizziness (PPPD) have been employed to describe nearly the same group of patients (4). It appears that all these syndromes represent a group with ill-defined borders. Hypersensitivity to visual stimuli and motion is also strongly associated to migraine and motion sickness (5), and thus, defining the extent of this phenomenon in chronically dizzy patients with a vestibular dysfunction is an important issue to consider.

Chronic dizziness is typically associated with anxiety and depression, but whether balance disorders are a consequence, or a contributing factor to these disorders remains unclear (3, 6). Psychological repercussions of long-term vertigo seem to extend beyond anxiety or depression and include depersonalization/derealization disorder (DRD) (7). Depersonalization is the subjective experience of detachment or estrangement from one's own self. Derealization is the equivalent subjective experience as applied to one's surroundings, animate or inanimate. Since these two experiences are often associated and there is no evidence to distinguish their nature, a single classification, namely DRD, has been adopted in DSM-5 (8). DD symptoms are typically observed in psychiatric illnesses, especially panic disorder and depression, and also in neurological disorders but may also represent a primary disorder (9, 10).

Depersonalization/Derealization (DD) symptoms as evaluated by DRD inventory (DDI) (11), are present in a higher proportion of individuals with vestibular disorders in comparison to healthy controls (6, 7, 12). Also, DDI scores appear to be related to anxiety and depression in patients with balance disorders (7). Finally, caloric vestibular stimulation increases the DDI scores in healthy adults (7) suggesting a direct link between vestibular inputs and DD symptoms.

Identifying subgroups of patients with chronic vertigo who are present DD symptoms will potentially lead to a better understanding of the phenomenon and targeted therapeutic actions. To our knowledge, the relation between visual and vestibular hypersensitivity (VVH), and DD symptoms has not been studied. We hypothesized that patients with chronic dizziness and VVH had higher anxiety, depression, and DDI scores.

The principal objective of this study was to investigate the relation between VVH and DD symptoms. In addition, we also analyzed the relation between several clinical parameters (especially age, sex, motion sickness, migraine, Hospital Anxiety, and Depression scale) and the extent of VVH and DD symptoms.

## Materials and Methods

This cross-sectional study included 319 consecutive patients with a spontaneous dizziness. The group was selected from a population of 500 consecutive patients examined for balance disorders in one tertiary referral center during 5 months. The study was conducted during a routine follow-up and data acquisition and analyses were not blinded. The inclusion criteria were: patients complaining of spontaneous dizziness according to International Classification of Vestibular Disorders I (ICVD-I V 1.0) (13) lasting for more than 3 months after the last acute episode of a possible triggering event, French-speaking patients capable of responding to questionnaires. Adults and teenagers were included regardless of their age. Two patients with bilateral vestibular loss were excluded due to possible confusion between symptoms due to the peripheral deficit and those related to central processing. Patients presenting with vertigo in addition to spontaneous dizziness were also excluded. The study was reviewed and approved by our institutional ethical committee (CPP Est III), and all patients provided their informed and written consent. The population comprised 214 females and 105 males with a mean age of 58 ± 17.4 years (range: 13–90 years). The mean delay between the triggering event and the inclusion was 4.1 ± 6.43 years. Initially, all patients underwent a thorough clinical examination, a caloric test, evaluation of oculomotricity, and subjective visual vertical. Based on this workup, 167 (52%) patients fulfilled the diagnostic criteria for persistent postural-perceptual dizziness [PPPD, (14)]: Unsteadiness > 3 months, exacerbation by upright position, self- or visual environment movements, significant functional handicap, and symptoms not better explained by any other disorder. This group included 125 women (74%) and 42 men (26%) with a mean age of 56 ± 17.4 years.

The possible triggering disease was classified into the following categories:

- Recent benign paroxysmal positional vertigo (3–12 months before inclusion, BPPV according to von Brevern et al. (15): *n* = 58 (18%)
- Cured BPPV (>12 months before inclusion): *n* = 85 (26%)
- Stress defined by anxiety or traumatic stress associated to spontaneous dizziness without abnormality of clinical and instrumental vestibular examination (16): *n* = 36 (11%)
- Probable Ménière's disease according to the Ménière's disease diagnostic criteria (17): *n* = 33 (10%)
- Vestibular migraine according to Barany Society criteria (18): *n* = 30 (9%)
- Otolithic dysfunction defined as vertigo or postural unsteadiness, normal canal function, and abnormality of sacculocolic or utriculoocular myogenic evoked potentials (19): *n* = 11 (3%)
- Unilateral vestibular loss defined by a canal paresis on bicaloric test (>30% asymmetry of the sum of 2 the stimulations measured by the slow-phase velocity of the nystagmus on videonystagmography) and video Head Impulse test (vHIT, gain < 0.7 on at least one canal on the same side): *n* = 8 (3%)
- Central disorders were defined as vertigo, dizziness, or unsteadiness associated to abnormal ocular pursuit control and/or gaze nystagmus and/or dysmetric saccades, and/or absent ocular fixation, and/or abnormalities of central vestibular pathways on MRI (20): *n* = 11 (3%)
- Age-related dizziness defined by age >75 years-old and spontaneous dizziness, and no evident deficit of canal or otolith function, and no identifiable neurologic abnormality: *n* = 8 (2%)
- Vestibular paroxysmia was diagnosed according to Strupp et al. (21). In this group patients were treated by carbamazepine with no acute vertigo: *n* = 3 (1%)
- Perilymphatic fistula diagnosed according to Portmann et al. (22). In this group patients were surgically treated with no more triggered vertigo or dizziness (13): *n* = 4 (1%)
- Drug-related group defined by patients with orthostatic dizziness, no clinical or instrumental signs of vestibular deficit and anti-hypertensive medication (23): *n* = 2 (1%).
- Undetermined triggering event: *n* = 30 (10%).

In addition to this routine workup, patients responded to 3 self-assessment questionnaires: Dizziness in Daily Activity (DDA), Hospital Anxiety Depression Scale (HAD) (24), and DDI (11).

In the DDA questionnaire, the patients were asked if they were dizzy in the 9 following situations: (1) Rapid head movements when dish-washing; (2) Sport and house-keeping; (3) Looking both ways before crossing the street; (4) Moving visual scene (e.g., crowd, traffic, malls, public transportation); (5) Climbing or coming down (e.g., stairs, pavement borders, bus); (6) Moving images on screens; (7) Undergoing acceleration and break (e.g., lift, car, train, speedwalk); (8) Bending forward (e.g., tying shoes, plugging a device, picking up an object from the ground); (9) Open spaces (e.g., parks, beaches, embankments). For each item, the patient indicated whether he/she was concerned by the activity and scored the dizziness from 0 (none) to 10 (maximal discomfort). A score of 11 was assigned to the activities which were avoided due to unbearable discomfort. A global score was calculated as the sum of the scores ranging from 0 to 99.

HAD scale comprised 14 questions pertaining to anxiety (*n* = 7) and depression (*n* = 7). Each item was scored from 0 to 3. A score was calculated for anxiety (aHAD) and depression (dHAD) separately ranging from 0 to 21. Characterized anxiety and depression were defined as a score > 8/21 for each subgroup of questions (24).

DDI included 28 questions. One point was assigned to each positive answer. The global score ranged from 0 to 28. Patients were asked to fill in the DDI concerning their status at the basal level and during a past vertigo spell. Information concerning migraine and motion sickness were also recorded.

### Statistical Tests

We tested the a priori hypothesis of a relationship between DDA score reflecting VVH in one hand and age, sex, the triggering disease, migraine, motion sickness, anxiety, depression, and DD symptoms on the other hand.

Data were analyzed by Statview (SAS Inc., Cary, NY). Values were expressed as mean ± SD. Continuous variables were analyzed by a paired or an unpaired *t-*test for 2 subgroups and ANOVA followed by a Bonferroni's test for multiple comparisons. A Mann-Whitney test was employed for the comparison of 2 groups when the normal distribution of the variable was not insured. A Fisher's exact test was used to compare categorical variables in 2 subgroups. *P* < 0.05 was considered as significant.

## Results

### Dizziness in Daily Activity Score

In total, 312 patients responded to the DDA questionnaire (Table 1). All the proposed items appeared to concern the majority of the patients. Women had a higher DDA score than males (25.6 ± 20.51, *n* = 210 vs. 15.2± 14.84, *n* = 102, *p* < 0.0001, unpaired *t-*test). The rate of avoidance was also higher in women (90 among 1,794 questions in women, 5% vs. 20 among 908 in men, 2%, *p* < 0.001, Chi-2 test). Age and etiology categories were similar in these subgroups and did not appear as confounding factors (data not shown).

**Table 1:** Dizziness in daily activity questionnaire.

**Items**	**Score**	**Not concerned (%)**	**Avoidance (%)**
Dish washing	1.4 ± 2.70 (277)	20	11
Sport and house-keeping	3.9 ± 4.06 (292)	10	40
Crossing street	2.1 ± 3.06 (299)	6	10
Crowd	3.3 ± 3.56 (304)	4	17
Stairs	2.8 ± 3.26 (304)	4	10
Screens	2.8 ± 3.53 (298)	6	19
Acceleration	2.6 ± 3.32 (302)	4	15
Bending forward	3.5 ± 3.36 (308)	2	9
Open spaces	0.9 ± 2.43 (283)	27	10
Global score	22.2 ± 19.45 (312)	

*The questionnaire was submitted to 319 patients. Patients rated their dizziness during the activity from 0 (none) to 11 (avoidance due to unbearable dizziness)*.

**Figure 1:**
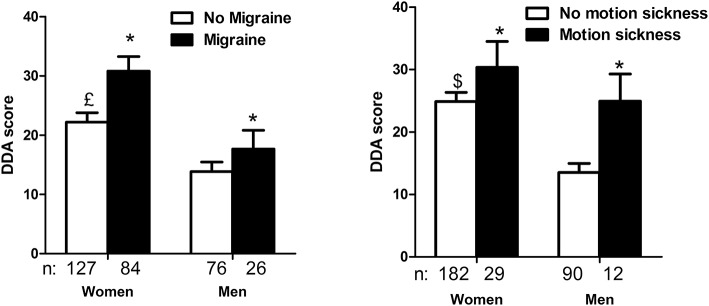
Dizziness in Daily Activity (DDA) scores as a function of sex, migraine history **(A)** and motion sickness **(B)**. Females had higher DDA scores than males. A personal history of migraine **(A)** and acquired motion sickness **(B)** also increased the scores without interaction with the effect of sex (^*^p < 0.05 for the effect of migraine, and motion sickness,^£^*p* < 0.0001 and ^$^*p* < 0.05 for the effect of sex, interaction not significant, 2-way ANOVA).

DDA score was also higher in migraineurs (27.9 ± 21.88, *n* = 111 vs. 19.1 ± 17.23, *n* = 201 patients without migraine, *p* < 0.001, unpaired *t-*test). The effect of migraine on the score appeared to be separate from the effect of sex (Figure 1A, *p* < 0.05 for the effect of migraine, *p* < 0.0001 for the effect of sex and no significant interaction, 2-way- ANOVA).

An acquired motion-sickness also appeared to increase DDA score (28.8 ± 20.45, *n* = 41 vs. 21.2 ± 19.08, *n* = 273 patients without motion sickness, *p* < 0.05, unpaired *t-*test). This effect appeared to be independent from the effect of sex (Figure 1B).

Age did not seem to influence the DDA score. Indeed, there was no correlation between age and DDA score (*R* = 0.08, simple regression analysis, not significant, ANOVA), and there was no difference of DDA scores between younger (<60 years) and senior patients (23.5 ± 20.65, *n* = 155 vs. 21 ± 18.2, *n* = 157, respectively, not significant, unpaired *t-*test). Similarly, etiology categories did not seem to influence DDA assessment (data not shown).

Forty-one patients (13%) declared no discomfort for all proposed activities (global score = 0). This group was composed of 20 males and 21 females with a mean age of 61 ± 16.1 years. This group was more masculine in comparison to the group scoring > 0 on DDA (49% vs. 30% of men, respectively, *p* < 0.05, Fisher's exact test). The group scoring 0 on DDA did not include any case of motion sickness while this symptom was noted in a significantly higher proportion of individuals among those with a DDA score above 0 (41 out of 273, *p* < 0.01, Fisher's exact test).

### Hospital Anxiety and Depression Scale

**Figure 2:**
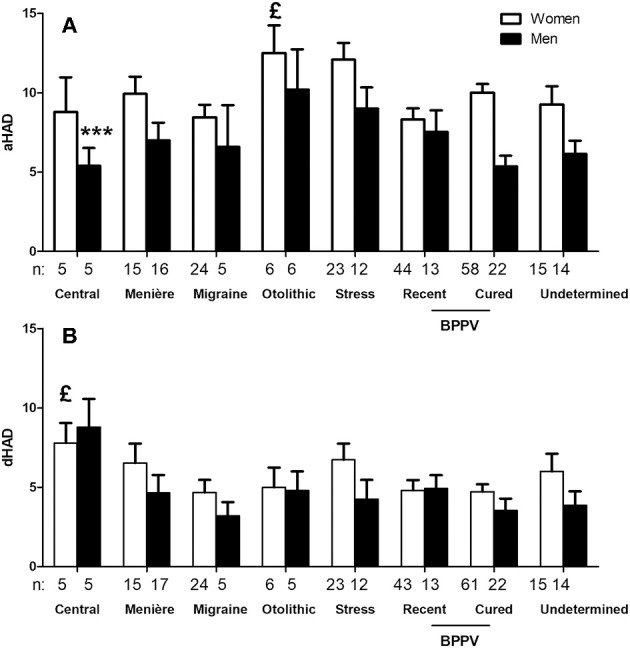
Hospital Anxiety and Depression (HAD) questionnaire anxiety scores as a function of sex and triggering disease. Only subgroups with *n* > 3 are represented. Sex and triggering disease both influenced the anxiety scores **(A)**,^£^*p* < 0.05 for the effect of triggering disease and ^***^p < 0.0001 for the effect of sex, not significant for interaction, 2-way ANOVA). Only etiology influenced depression scores **(B)**,^£^*p* < 0.05 for the effect of triggering disease and not significant for the effect of sex, 2-way ANOVA). Central dizziness had higher depression scores (*P* < 0.001, Bonferroni post-test).

Anxiety (aHAD) score in our population with chronic dizziness was 8.6 ± 4.54 (*n* = 305).This score was higher in women (9.5 ± 4.42, *n* = 203 vs. 6.9 ± 4.24, *n* = 102 for men, *p* < 0.0001, unpaired *t-*test) and in young patients (9.4 ± 4.45, *n* = 153 in patients <60 years vs. 7.8 ± 4.50, *n* = 152 in > 60 years, unpaired *t-*test, *p* < 0.01). Etiology also seemed to influence the anxiety scores. Higher scores were recorded in stress-related dizziness and otolithic syndrome (Figure 2).

Patients with a history of migraine reported higher anxiety levels than others (9.7 ± 4.64, *n* = 106 vs. 8.1 ± 4.37, *n* = 199, respectively, *p* < 0.01, unpaired *t-*test). Similarly, those with acquired motion sickness had higher aHAD ratings (9.5 ± 4.52, *n* = 39 vs. 8.5 ± 4.53, *n* = 266, *p* < 0.05, unpaired *t-*test).

Depression (dHAD) score in the population was 5.0 ± 3.94 (*n* = 308). There was no difference between men and women (4.5 ± 3.71, *n* = 104 vs. 5.3 ± 4.05, *n* = 204 not significant, unpaired *t-*test). In the same manner as aHAD, scores in young patients tended to be higher than those in senior patients (5.3 ± 4.08, *n* = 153 vs. 4.6 ± 3.77, *n* = 155, respectively, mean difference = 0.7, *p* = 0.08, unpaired *t-*test). dHAD did not seem to be influenced by a history of migraine or acquired motion sickness (data not shown).

**Figure 3:**
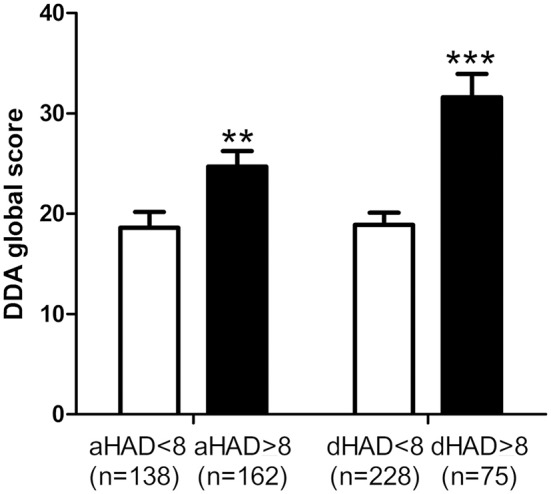
Relation between Dizziness in Daily Activity (DDA) and Hospital Anxiety and Depression (HAD) scores. Subjects with a characterized anxiety (anxiety score on HAD, aHAD > 8) and depression (depression score on HAD, dHAD > 8) had higher DDA scores. Bars represent mean ± SEM. ^**^p < 0.01, ^***^p < 0.001, unpaired *t*-test.

Patients with characterized anxiety or depression (scores > 8) had higher DDA scores than those with scores below 8 (Figure 3), suggesting a relation between daily activity discomfort and anxiety/depression levels.

### Depersonalization/Derealization Inventory

**Figure 4:**
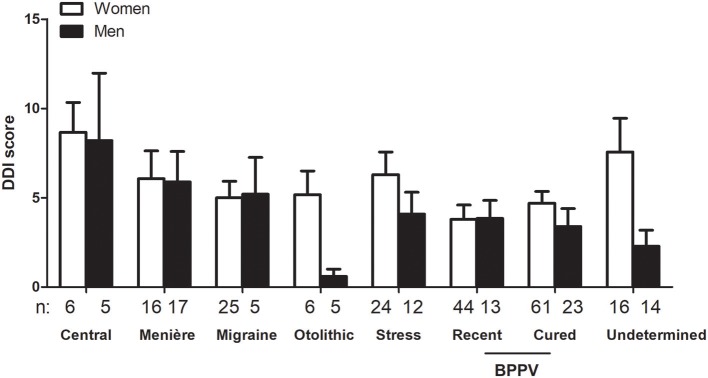
Influence of sex and triggering disease on Depersonalization/Derealization Inventory (DDI) scores. Sex and triggering disease both influenced DDI scores (*p* < 0.05 for the effect of sex and *p* < 0.05 for the effect of etiology, not significant for interaction, 2-way ANOVA). Only triggering disease groups with *n* > 3 for men and women are represented. Bars represent mean ± SEM. BPPV: Benign paroxysmal positional vertigo.

The average score for DDI was 4.7 ± 5.28 (*n* = 318). Women scored higher than men (5.1 ± 5.42, *n* = 213 vs. 3.9 ± 4.91, *n* = 105, respectively, *p* < 0.05, unpaired *t-*test). Also, patients below 60 years had higher scores than seniors (5.4 ± 6.04, *n* = 156 vs. 4.0 ± 4.35, *n* = 162, respectively, *p* < 0.05, unpaired *t-*test). As for the HAD questionnaire, etiology also affected DDI score independently from the sex with higher levels in central disorders and in women (Figure 4).

Patients with a personal history of migraine also provided higher DDI ratings in comparison to those without a migraine history (6.1 ± 6.40, *n* = 110 vs. 4.0 ± 4.42, *n* = 210, respectively, *p* < 0.001, unpaired *t-*test). Patients with acquired motion sickness had also higher DDI scores than those who did not suffer from it (6.8 ± 5.93, *n* = 41 vs. 4.4 ± 5.11, *n* = 279, respectively, *p* < 0.01, unpaired *t-*test).

Patients with characterized anxiety and depression (aHAD and Dhad > 8) had higher DDI scores (6.0 ± 5.75, *n* = 167 for aHAD ≥ 8 vs. 3.0 ± 4.11, *n* = 139 for aHAD< 8, *p* < 0.0001, and 7.6 ± 6.66, *n* = 79 for dHAD ≥ 8 vs. 3.6 ± 4.33, *n* = 230 for dHAD< 8, *p* < 0.0001, unpaired *t-*test).

The analysis of the parameters which influenced DDA score in our population by a multiple regression model showed that the combination of dHAD, aHAD, DDI, and age as independent factors were correlated to DDA (Adjusted *R* = 0.52, *p* < 0.0001, ANOVA, *n* = 296).

**Figure 5:**
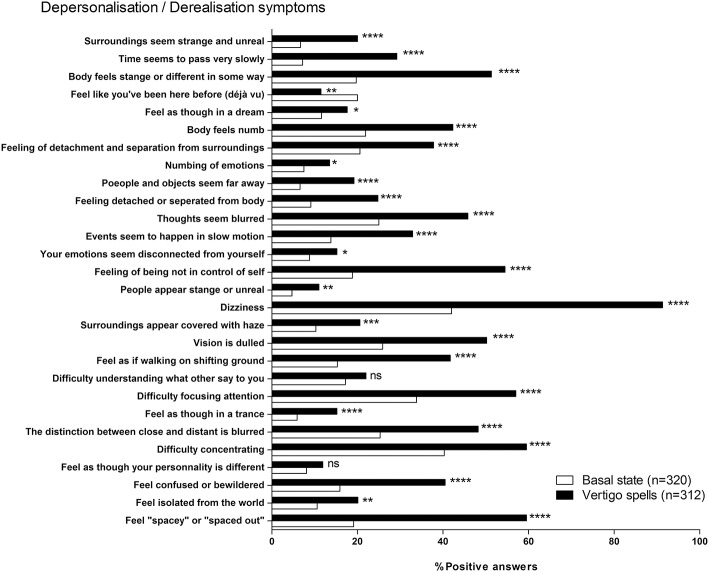
Depersonalization/Derealization Inventory (DDI) at basal state and during vertigo spells. ^*^p < 0.05, ^**^p < 0.01, ^***^p < 0.001, and ^****^p < 0.0001, ns, not significant; Khi-2 test vs. basal state.

**Figure 6:**
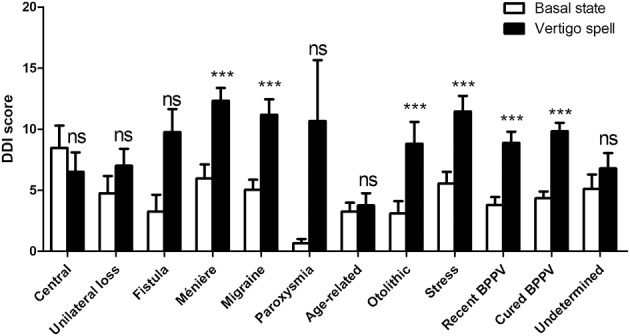
Depersonalization/Derealization Inventory (DDI) scores at basal state and during vertigo spells as a function of triggering disease. Both triggering disease and vertigo spells influenced the score (*p* < 0.0001 for the effect of vertigo spells, not significant the effect of triggering disease and for interaction, 2-way ANOVA). Bars represent mean ± SEM. Ns, not significant, ^***^p < 0.001, Bonferroni's post-test vs. basal state. BPPV, Benign paroxysmal positional vertigo.

When asked to score their perception during past vertigo spells by the DDI, patients estimated their DD symptoms higher than the one at the basal state (9.6 ± 6.67, *n* = 312 vs. 4.7 ± 5.28, *n* = 318, respectively, *p* < 0.0001, paired *t-*test). This significant increase concerned 25 items out of 28 (Figure 5). The mean variation of the score was 4.9 ± 5.98 (*n* = 311, range: −8 to 26). Younger patients (<60 years) had higher DDI score shifts during the spells than the seniors (6.4 ± 6.22, *n* = 154 vs. 3.5 ± 5.40, *n* = 157, *p* < 0.0001, unpaired *t-*test). But the sex did not seem to influence the amplitude of the shift (4.5 ± 5.60, *n* = 103 for men vs. 5.0 ± 6.14, *n* = 207 for women, not significant, unpaired *t-*test). History of migraine and acquired motion sickness did not influence the amplitude of the shift (5.4 ± 6.47, *n* = 107 for migraineurs vs. 4.6 ± 5.67, *n* = 203 for non-migraineurs, and 5.0 ± 6.09, *n* = 270 for motion sickness vs. 4.4 ± 5.00, *n* = 40 for no motion sickness, not significant, unpaired *t-*test). Etiology did not seem to alter the DDI score shift during spells (Figure 6).

## Discussion

In this study, we showed that patients with chronic balance disorder complaining from vestibular and visual discomfort in daily life activities were frequently young women with a history of migraine or acquired motion sickness. The extent of the discomfort was related not only to depression and anxiety levels, but also to the depersonalization/derealization sensations. The intensity of these latter increased during vertigo spells suggesting the impact of vertigo on the perception of self, environment, and time.

The relation between anxiety, depression, and vertigo has been already reported in several studies (25). This association is probably explained by the close connections between the vestibular and the limbic systems (26). In our study, other symptom related to the triggering event such as headaches, tinnitus, and hearing loss could also interfere with anxiety and depression, or even depersonalization/derealization. However, it is very difficult to estimate this participation. Vertigo decreases in the majority of cases, even if the initial disorder persists (e.g., vestibular neurectomy), via a neuronal reorganization at the level of the brainstem but also thalamic and cortical centers leading to the central vestibular compensation (27). However, if the vestibular function fluctuates, or if the rehabilitation exercises are insufficient, monotonous, or late the central process cannot accomplish a complete compensation (27). Interestingly, psychological factors seem also crucial in the compensation: passivity, depression, and avoidance largely influence balance performances (27). Our results suggest that other subgroups of patients may also encounter difficulties to compensate their vestibular dysfunction. Female patients appeared to present with more vestibular and visual discomfort than males independently from other possible confounding factors (age, migraine, motion, sickness).

An extensive literature has suggested the sex difference in the integration of visual inputs into the balance function and motion sickness (28, 29). Women are more frequently subject to motion sickness and exhibit higher scores at motion sickness susceptibility questionnaire (29). This susceptibility seems to decrease in senior patients (29). In addition, posturography shows that women tend to couple their sway to the moving environment in a less extent than men (28). Although, the explanation for this difference is unclear, the influence of sex hormones on the fluctuation of vestibular function could explain the pronounced discomfort in female patients (30).

The same female preponderance has been observed for the incidence of migraine (31). In this disease, the role of estrogen has been well-documented (31). A close relationship between migraine, motion sickness, and vestibular disorders has also been established. In a recent study, Ghavami et al. showed that migraine, sensitivity to visual motion, light and sound, head motion, smells, weather changes, or medication was present in 95% of all patients with definite Menière disease and that this population was predominantly feminine (70%) (32). These observations are in accordance with our results showing higher vestibular and visual discomfort in females, in migraineurs and in those suffering from motion sickness.

Current questionnaires evaluating vertigo such as Dizziness Handicap Inventory do not assess VVH (33). Situational Characteristics Questionnaire (SCQ) focuses more on VVH and is validated in Canadian French (34). However, its use in France would have necessitated adaptation and validation since the two languages and every-day life habits are different. While car and bus trips are very detailed, many other situations which we explore are not taken into account (e.g., screens, dish-washing). Moreover, patients who are not concerned by the proposed activities are not recorded and the avoidance due to extreme stress is not considered in SCQ.

Consequently, to the aim of investigating the relation between VVH and DD symptoms, we designed an in-house questionnaire especially targeting situations in which VVH is incapacitating and assessed the handicap by a Likert scale. The validity of the Likert method as a psychometric tool has been demonstrated in many domains specially in chronic vertigo (35) The addition of avoidance as an indicator of extreme handicap in some activities appeared to us as crucial. Likert scales remain valid with 11 levels (36). However, this questionnaire needs to be further investigated for validity and reliability.

The relation between self-awareness and vestibular function was investigated as early as the beginning of twentieth century (37). Since its first description, this relation has been largely studied with complex experimental paradigms in normal subjects and in patients with vestibular loss (38). Vestibular stimulations modulate the sense of owning a body and anchors the self to the body (39). Negative emotions enhance self-motion detection (40).

The relation between DD symptoms and vestibular disorders has been previously reported by Sang et al. (7). These authors investigated the basal DD symptoms level and the effect of a caloric vestibular stimulation in healthy subjects and in patients with peripheral vestibular disorders (unilateral canal paresis, BBPV) by the DDI. This study showed that DD symptoms were more intense in patients with a vestibular disorder (with and without recent symptoms) than in normal subjects. Patients with recent vestibular symptoms had also higher DDI scores than those with past symptoms. They also observed that DDI scores increased during a vestibular caloric test in normal individuals. These results are in accordance with our observations and suggest a strong link between the vestibular network and the centers regulating the self-awareness. In addition to the previously reported results, we showed that women had tendency to score higher on DDI. Similarly, patients below 60 years of age, those suffering from migraine, and motion sickness reported higher DDI scores. This observation provides a possible link between the above-mentioned observations on migraine, motion sickness, vestibular disease, and the possible role of sex hormones. We also showed that DDI scores increase during the past vertigo spells. This result is also in accordance with the increase of DDI in normal individuals during caloric stimulation (7). The amplitude of the score shift during the spells was significantly greater in young patients. This information underlines the relation between the vestibular input and the perception of the environment and the self.

In conclusion, chronic dizziness can entail not only anxiety and depression but also sensations of depersonalization, and derealization independently from the etiology. The observation that DDI scores increase during vertigo spells suggests that balance disorders enhance depersonalization and derealization. This possible causality can be explained by the disturbances of our internal body scheme and the environment representation during vestibular disorders, and the uncertainty on the validity of sensory inputs that they generate. DD complaints were more frequent and intense in young female patients and in those suffering from migraine and motion sickness. These patients also reported incapacitating symptoms related to visual and vestibular hypersensitivity.

## Data Availability Statement

The raw data supporting the conclusions of this manuscript will be made available by the authors, without undue reservation, to any qualified researcher.

## Author Contributions

MT and AB analyzed data and prepared the manuscript. CV, CH, and UD participated in the study design. MT, AC, and SH included and examined the patients.

### Conflict of Interest Statement

The authors declare that the research was conducted in the absence of any commercial or financial relationships that could be construed as a potential conflict of interest.
